# Clinical Use of PPAR*γ* Ligands in Cancer

**DOI:** 10.1155/2008/159415

**Published:** 2008-12-18

**Authors:** Jennifer L. Hatton, Lisa D. Yee

**Affiliations:** Division of Surgical Oncology, Department of Surgery, The Ohio State University, Columbus, OH 43210, USA

## Abstract

The role of PPAR*γ* in adipocyte differentiation has fueled intense interest in the function of this steroid nuclear receptor for regulation of malignant cell growth and differentiation. Given the antiproliferative and differentiating effects of PPAR*γ* ligands on liposarcoma cells, investigation of PPAR*γ* expression and ligand activation in other solid tumors such as breast, colon, and prostate cancers ensued. The anticancer effects of PPAR*γ* ligands in cell culture and rodent models of a multitude of tumor types suggest broad applicability of these agents to cancer therapy. This review focuses on the clinical use of PPAR*γ* ligands, specifically the thiazolidinediones, for the treatment and prevention of cancer.

## 1. INTRODUCTION

The peroxisome proliferator-activated receptor *γ* (PPAR*γ*) is a ligand-activated transcription factor in the superfamily of the steroid/thyroid nuclear hormone receptors, one of three PPAR isotypes (*α*, *β*/*δ*, and *γ*) [1–3]. PPARs share a highly conserved DNA-binding domain that matches with specific DNA sequences known as peroxisome proliferator response elements (PPREs), binding as a heterodimer with retinoid X receptor (RXR) to initiate transcription of target genes [4–6]. Highly expressed in adipose tissue, PPAR*γ* is best known for its important role in adipocyte differentiation [7–9].

PPARs were first shown to be activated by compounds inducing peroxisome proliferation [10, 11], then by a variety of polyunsaturated fatty acids (PUFAs) in the micromolar range [12]. PPAR*γ* has a more restricted list of activators compared to the other two isotypes, being more selective to PUFAs compared to other fatty acids [13, 14]. Fatty acid derivatives, such as 15-deoxy-Δ^12,14^-PGJ_2_ (15d-PGJ_2_) and 13- and 9-hydroxyoctadecadienoic acid (HODE), have also been identified as activators of PPAR*γ* [15–17]. Synthetic ligands for PPAR*γ* include the thiazolidinediones, a class of oral hypoglycemic drugs that reduce hyperglycemia and hyperinsulinemia in insulin-resistant states [18]. Nonsteroidal anti-inflammatory drugs (NSAIDs) such as ibuprofen are also PPAR*γ* ligands, but exhibit much lower binding affinities in comparison to thiazolidinediones [19].

A wealth of preclinical data supports a role for PPAR*γ* ligand therapy in many different types of solid malignancies. Experimental studies of human cancer cells and PPAR*γ* have focused primarily on the *γ*-activating effects of thiazolidinediones, identifying PPAR*γ* agonists as negative regulators of cell growth and tumor progression. As a putative natural ligand for PPAR*γ*, 15d-PGJ2 also appears to have anticancer effects. Treatment with PPAR*γ* ligands inhibits malignant cell proliferation, with evidence for cell cycle arrest and induction of apoptosis [20–25]. With the pivotal role of PPAR*γ* in adipocyte differentiation, PPAR*γ* ligands have been tested for differentiating effects on malignant cells. Changes consistent with induction of a more differentiated cancer cell phenotype were detected in several tumor model systems, including breast, colon, and liposarcoma [9, 26–30].

The anticancer effects of PPAR*γ* agonists may also be mediated in part via suppression of an angiogenic tumor phenotype or angiogenic, inflammatory tumor microenvironment. PPAR*γ* ligands appear to elicit antiangiogenic effects, via modulation of endothelial cell function and growth [31–35]. The anti-inflammatory actions of PPAR*γ* ligands [36–38], which are of relevance to the treatment of atherosclerosis and cardiovascular disease, may also prove important for the treatment and prevention of cancer given the association of chronic inflammation with increased cancer risk [39].

Approved by the Food and Drug Administration for treatment of type 2 diabetes mellitus, thiazolidinediones have been evaluated for use as investigational cancer therapies. The initial studies utilized troglitazone (Rezulin), which was the first thiazolidinedione in clinical use but ultimately withdrawn from the US market in 2000 because of instances of severe idiopathic liver disease [40]. Rosiglitazone (Avandia) and pioglitazone (Actos) were thereafter used without evidence of similar hepatotoxicity. The majority of clinical trials of thiazolidinediones have been conducted in advanced stages of disease. The following review examines the clinical trial experience to date with thiazolidinediones in cancer.

## 2. MONOTHERAPY

Searches of the PubMed and www.clinicaltrials.gov databases identified nine clinical trials testing the efficacy of thiazolidinediones as single agent therapy in cancer patients, with the majority conducted in subjects with advanced and/or metastatic stages of disease refractory to treatment. Troglitazone was the drug of choice for human studies initiated prior to 2000, replaced by rosiglitazone or pioglitazone in trials conducted after the withdrawal of troglitazone from clinical use because of rare instances of hepatotoxicity. Thiazolidinediones have been generally administered at the highest recommended dose for treatment of diabetes mellitus, or slightly higher as with troglitazone at 800 mg/day. Rather than mimic the generalized cytoxicity of chemotherapeutic regimens, such doses might selectively target PPAR*γ*-mediated effects such as cellular differentiation, growth inhibition, and induction of apoptosis.

### 2.1. Liposarcomas

As mesenchymal malignancies that arise from adipose tissue [41, 42], liposarcomas express PPAR*γ* at levels comparable to normal adipose tissue [26]. PPAR*γ* expression is a distinguishing feature of liposarcomas relative to other soft tissue sarcomas [43]. Treatment of primary cell strains derived from human liposarcomas (two well-differentiated liposarcomas and one intermediate grade myxoid/round cell liposarcoma) with PPAR*γ* and/or RXR ligands induced morphologic and molecular changes consistent with adipocyte differentiation [26]. These in vitro findings led to consideration of thiazolidinediones for differentiation therapy of liposarcomas.

Demetri et al. conducted an open label phase II clinical trial to determine the effects of troglitazone (800 mg/day) on tumor differentiation in patients with advanced and/or metastatic liposarcoma [44]. In a report of three study subjects, tumor samples obtained after the six week intervention showed marked cytoplasmic lipid accumulation in comparison to pretreatment biopsies, as well as morphologic changes suggestive of mature adipocytes. The expression of markers of adipocyte differentiation such as adipsin, aP2, and PPAR*γ* by Northern analysis increased following treatment with troglitazone. Furthermore, cell proliferation as assessed by the percentage of liposarcoma cells with Ki67 immunostaining decreased by two- to fourfold with the study intervention. Three additional patients enrolled in the study also had post-treatment biopsies with histologic evidence of adipocyte differentiation and decreased cell proliferation. The histologic subtypes treated in this study ranged from intermediate to high grade, and the authors raise the possibility that differentiation may prove difficult to assess in more differentiated tumors. This small pilot trial of six subjects demonstrated a differentiating effect of short-term troglitazone therapy in advanced stage liposarcomas providing the rationale for further clinical evaluation of troglitazone and other PPAR*γ* ligands in the management of liposarcomas.

A second phase II trial evaluated the effects of the thiazolidinedione rosiglitazone (8 mg administered as 4 mg twice daily) in nine patients with advanced, unresectable liposarcomas [45]. Analyses of tumor samples obtained at 0, 6, and 12 weeks did not reveal changes in histopathology or gene expression to support a differentiating effect. By quantitative RT-PCR, levels of adipsin, fatty acid-binding protein (FABP), and PPAR*γ* increased in only two of nine tumors although in a somewhat inconsistent fashion. In one of the patients with a dedifferentiated liposarcoma, expression of PPAR*γ* increased at week 12, FABP at week 6 and adipsin at weeks 6 and 12. However, disease progression had been detected in this patient at 10 weeks of treatment, such that the expression of differentiation markers did not correlate with clinical benefit. For the other subject, upregulation of adipsin at week 12 was noted in a myxoid liposarcoma, and treatment continued until progression of disease at six months. Cell proliferation assessed by Ki67 immunostaining did not decrease significantly with the study intervention, although the low baseline levels of proliferation decreased further with rosiglitazone. Taken together the data fail to support a role for rosiglitazone in the treatment of liposarcoma at advanced stages.

The discrepant findings between the two phase II trials of thiazolidinedione therapy may relate to differences in the patient cohorts, tumors, and/or study agents. Whether methodological issues contributed to the differences is not clear. As surgery remains the mainstay of treatment for liposarcomas, with limited therapeutic options for unresectable, advanced disease, and investigation of PPAR*γ* ligands for adjuvant therapy of liposarcomas or control of microscopic or minimal residual disease may still be warranted.

### 2.2. Colon cancer

Considerable in vitro evidence exists to support the differentiating and antiproliferative effects of PPAR*γ* ligand therapy in colon cancer. PPAR*γ* is highly expressed in human colon tumor specimens and cancer cell lines [27, 46]. In addition to growth inhibition, treatment of colon cancer cells with PPAR*γ* ligands promotes tumor cell differentiation as assessed by increased cytoplasmic to nuclear ratio and higher levels of differentiation markers such as carcinoembryonic antigen (CEA), villin, intestinal alkaline phosphatase, GOB-4, and keratin 20 [27, 29, 47]. Although in vitro and in vivo studies demonstrate a role for PPAR*γ* in modulating growth and differentiation of human colon cancer cell lines [27], thiazolidinedione treatment with rosiglitazone (BRL49653) or troglitazone stimulated rather than inhibited the development of colon tumors in APC^min/+^ mice [48, 49]. The loss of adenomatous polyposis coli (APC) tumor suppressor gene function, which leads to accumulation of *β*-catenin and enhanced Wnt pathway signaling, may account for the contradictory findings of enhanced tumorigenesis in this genetic versus xenograft model of colon cancer [50]. Interestingly, treatment with pioglitazone over a wide range of doses suppressed intestinal tumor formation and hyperlipidemia in APC deficient mice (APC^min/+^ and Apc1309) [51, 52]. Whether the use of different thiazolidinediones or experimental conditions led to the discrepant findings is unclear.

Loss of function mutations in *PPARG* in colon cancer may also account for variability in response to PPAR*γ* therapy. Four unique mutations of PPAR*γ* were identified in 55 sporadic colon cancers in exons 3 or 5; one yielding a truncated protein with loss of the ligand binding domain (c.472delA) and three causing defects in the binding of synthetic or natural ligands (Q286P, K319X, and R288H) [53]. A point mutation in exon 6, K422Q, was detected in four human colon cancer cell lines that were resistant to the antiproliferative and differentiating effects of PPAR*γ* ligands [54]. However, K422Q may have limited clinical relevance, as a study of 170 primary human colorectal cancers and 12 liver metastases failed to detect the presence of K422Q mutations [55].

A phase II trial evaluated troglitazone therapy (800 mg/day as a single dose) in 25 colon cancer patients with lung and/or liver metastases [56]. Although well-tolerated, troglitazone did not elicit objective tumor responses. All patients had progression of disease with a median progression-free survival of 1.6 months and median overall survival of 3.9 months. Troglitazone does not seem to have activity in advanced colon cancer, although the lack of response may relate at least in part to the biologically aggressive nature of Stage IV colon cancer that is refractory to cytotoxic chemotherapy (the median number of prior chemotherapy regimens was 3). Although the study was not designed to assess troglitazone for possible tumor promoting effects, Kulke et al. urged caution in the use of PPAR*γ* agonists in colon cancer. In the future, advances in the genomic profiling of colon cancer might allow for identification of susceptible colon cancer subtypes.

Colorectal cancer provides an excellent model of multistep progression in carcinogenesis, based on the acquisition of biochemical, molecular, and genetic alterations that transform normal epithelium to adenomatous polyps to invasive cancer [57]. PPAR*γ* ligands may have a role in chemoprevention of colon cancer, depending on the specific molecular and genetic subtype. Thiazolidinediones suppress the development of aberrant crypt foci (ACF), a putative precancerous lesion of the colon [58]. However, activation of certain oncogenic signaling pathways at later stages of carcinogenesis may override this regulatory role, as suggested by the APC^min/+^ mouse studies [50]. Timing of PPAR*γ* therapy prior to certain initiating events in colon carcinogenesis may thus prove to be useful for chemoprevention of colon cancer. As suggested by the in vivo studies of thiazolidinediones in min mice, use of PPAR*γ* agonists in colon cancer prevention may require identification of high-risk individuals based on genetic susceptibility as well as aberrant gene expression and signal transduction.

Although the differentiating and antiproliferative effects of PPAR*γ* activation form the basis for evaluating PPAR*γ* ligands for cancer therapy, the anti-inflammatory effects of PPAR*γ* may also be relevant to colon carcinogenesis. Chronic inflammation is linked to carcinogenesis, and diagnoses of inflammatory bowel disease (IBD), which includes Crohn's disease and ulcerative colitis, carry increased risk for colorectal cancer [59]. PPAR*γ* ligands repress cytokine production by colon cancer cells and inhibit chemically-induced colitis in murine models of inflammatory bowel disease [60–62]. These findings led to an open-label pilot trial of rosiglitazone therapy (4 mg taken twice daily) in patients with active ulcerative colitis for 12 weeks, showing clinical improvement in 8/15 (53%) patients of which 4 (27%) subjects had clinical remission [63]. Two subjects required hospitalization for worsening disease; other serious adverse events included severe pharyngitis requiring hospitalization and nephritic syndrome leading to withdrawal. The increased risk for colon cancer in patients with ulcerative colitis relates to the severity and duration of disease; by suppressing inflammation in the colon, drugs such as the thiazolidinediones or other PPAR*γ* agonists might play a role in colon cancer prevention.

### 2.3. Breast cancer

In vitro and in vivo research studies support a role for PPAR*γ* agonists in breast cancer therapy. Thiazolidinedione treatment of human breast cancer cells appears to induce changes suggestive of terminal differentiation, resulting in the inhibition of cell proliferation, suppression of keratin 19, mucin-1, and upregulation of maspin [28]. Troglitazone also inhibited breast cancer growth in a xenograft animal model [20]. Another synthetic ligand of PPAR*γ*, GW7845, had inhibitory effects in a carcinogen-induced model of mammary tumorigenesis [64]. Furthermore, PPAR*γ* haploinsufficiency (PPAR*γ*+/−) conferred increased susceptibility to dimethylbenz[a]antracene (DMBA)-induced mammary carcinogenesis [65]. These studies suggest that PPAR*γ* ligands could serve as negative regulators of breast cancer development and progression.

We and others have demonstrated PPAR*γ* immunostaining in human breast cancer specimens, as well as normal and benign proliferative breast tissue [28, 66]. PPAR*γ* appears to be expressed in most if not all breast cancer cell lines, as well as in normal and malignant breast tissue [20, 28, 66]. We did not find that the invasive, metastatic phenotype of the breast cancer cells correlates with PPAR*γ* expression levels, gel shift mobility patterns, or relative sensitivity to PPAR*γ* ligands [67]. All of the cell lines tested (MCF-7, MDA-MB-436, MDA-MB-231, and MDA-MB-435) responded to thiazolidinedione treatment in transactivation assays, which also led to inhibition of cell growth.

However, a Phase II trial of troglitazone in metastatic breast cancer refractory to cytotoxic chemotherapy did not demonstrate beneficial effects on disease progression [68]. In twenty two Stage IV breast cancer patients with an 8-week median treatment duration, troglitazone at 800 mg/day did not prevent disease progression. The median time on study was 56 days, ranging from 11 to 134 days; 19/22 subjects were removed from the study for objective or subjective evidence of disease progression. There were no objective responses, and only 3/21 (14%) subjects completing 8 weeks of troglitazone therapy had stable disease. In patients with elevated serum levels of CEA and/or CA27.29 at the onset of the study, these tumor markers had continued to increase despite 8 weeks of troglitazone. Burstein et al. note that more selective criteria to target PPAR*γ* therapy to the most susceptible tumor type might be helpful. The lack of effect might have also related to an advanced stage of disease that had become refractory to cytotoxic therapies.

To assess the effects of PPAR*γ* ligand therapy on breast cancer, we conducted a pilot study of short-term administration of rosiglitazone (4 mg taken twice a day) to women newly diagnosed with early stage breast cancer (Stages 0 to II) during the two- to six-week period between diagnostic biopsy and surgical removal of the cancer [69]. Thirty eight women completed the study intervention without serious adverse events; thirty four subjects had sufficient pre and posttreatment tumor tissue for correlative analyses. By Ki67 immunostaining, short-term intervention with rosiglitazone did not lead to statistically significant differences cell proliferation. We did not detect any somatic or germline mutations in *PPARG* to account for differential tumor tissue responses or lack of effect. Comparing H&E-stained sections of tumor before and after rosiglitazone therapy also failed to note increased tumor cell differentiation by standard criteria of tubule formation, mitotic activity, and nuclear morphology. PPAR*γ* expression in tumor samples was graded 0 to 3+ for intensity of nuclear and cytoplasmic immunostaining, which did not reflect an overall change in expression following rosiglitazone therapy. Interestingly, nuclear expression of PPAR*γ* was significantly reduced in a majority of the patients whose pre-rosiglitazone PPAR*γ* scores were 1+ or 2+ and therefore assessable for increased or decreased levels of expression, which raised the possibility of a PPAR*γ* mediated response in the tumor tissue.

Serum levels of adiponectin and indices of insulin sensitivity increased with rosiglitazone, based on serum samples obtained from seventeen subjects. Given the association of lower levels of adiponectin and insulin resistance with increased breast cancer risk [70–73], therapy with rosiglitazone or other PPAR*γ* ligands may have a role in breast cancer prevention. Of interest are potential effects of rosiglitazone therapy on nonepithelial components of the mammary tumor microenvironment; unfortunately, pretreatment core biopsy samples in our study had negligible to minimal stromal, noncancerous tissue for any comparative analyses.

Although short-term intervention with rosiglitazone did not alter the endpoint of cell proliferation, this pilot study indicated the potential for other tumor tissue specific effects with evidence for downregulation of PPAR*γ* protein expression. Breast cancer is a family of diseases with diverse molecular, genetic features, and PPAR*γ* ligands may affect only certain subtypes. Indeed, breast cancer therapy already encompasses molecularly targeted strategies, such as tamoxifen for estrogen receptor positive cancer and trastuzumab for tumors with HER-2/neu overexpression. Variability in PPAR*γ*-mediated effects on cell proliferation could arise from tumor heterogeneity in nuclear receptor cross-talk, complement of cofactors, or mutated or aberrant signaling pathways that override PPAR*γ* signaling, leading to an apparent lack of effect of rosiglitazone [74–77]. For example, in vitro studies indicate that estrogen receptor *α* (ER*α*) can repress PPAR*γ* signaling by binding to PPREs and that PPAR/RXR heterodimers can competitively inhibit ER binding at specific estrogen response elements (EREs) [74, 75]. In vitro administration of the PPAR*γ* ligand 15-deoxy-Δ12,14-prostaglandin J2 inhibited estrogen-mediated transactivation of ERE and estrogen-responsive gene expression in MCF-7 breast cancer cells [78]. By immunohistochemical analysis, PPAR*γ* expression has been detected in 42% (99/238) to 58% (101/170) of human breast cancer samples, correlating positively with ER expression and improved clinical outcome [78, 79]. Concomitant administration of other drugs, such as estrogen in postmenopausal women or thyroid hormone in patients with hypothyroidism, may therefore affect the interplay between nuclear receptors. Our sample size did not support subset analyses, and further studies are needed to identify specific molecular subtypes of breast cancer that are susceptible to PPAR*γ* ligand therapy and crosstalk interactions. These data also raise the possibility of PPAR*γ*-mediated modulation of systemic conditions relevant to breast cancer risk, such as serum adiponectin and insulin resistance.

Breast cancer treatment and prevention may benefit from future studies of PPAR*γ* therapy that address issues of susceptible breast cancer subtypes, duration and timing of intervention in the multistep process of mammary carcinogenesis. Combination of PPAR*γ* ligands with other agents may enhance therapeutic efficacy, as with RXR ligands for synergism in PPAR*γ*-RXR heterodimer-mediated signaling [80]. Interestingly, inhibition of HER-2/neu tyrosine kinase activity in a prostate cancer model prevents PPAR*γ* degradation and thereby enhances susceptibility to PPAR*γ* activators such as R-etodolac [81]. Administration of a PPAR*γ* agonist in conjunction with trastuzumab (Herceptin), a humanized monoclonal anti-HER-2/neu antibody used in the treatment of metastatic and high-risk HER-2/neu+ breast cancer, could also represent a novel combination of targeted therapies.

### 2.4. Prostate cancer

PPAR*γ* is expressed in human prostate cancer cell lines and human prostate cancer specimens [82]. In vitro and in vivo studies also demonstrate the anticancer effects of PPAR*γ* ligands on prostate cancer, including the reduction of prostate specific antigen (PSA) levels in androgen responsive LNCaP cells [82–85].

A phase II clinical study assessed the effects of troglitazone at 800 mg/day in 41 men with androgen-dependent (*n* = 12) or androgen-independent prostate cancer (*n* = 29) that had progressed following local treatment or androgen deprivation therapy yet remained asymptomatic [82]. Four patients with androgen-dependent cancers had a reduction in serum PSA levels after onset of therapy, which were measured every 4 weeks, with a greater than 50% decrease in this tumor marker detected in one patient after 16 months of troglitazone. PSA levels decreased less than 50% in four patients with androgen-independent prostate cancer. The median duration of troglitazone treatment was longer in the androgen dependent (26.8 weeks) versus independent (14.3 weeks) group and could correlate with the more indolent course of disease associated with the former, as well as greater susceptibility of this prostate cancer subtype. As suggested by Mueller et al., variability in response might relate to activated mitogen-activated protein (MAP) kinase signaling leading to phosphorylation and inactivation of PPAR*γ* as well as the possibility of somatic *PPARG* mutations.

Following from this initial study, a randomized placebo-controlled Phase II trial of rosiglitazone (8 mg/day) was conducted in prostate cancer patients with progressive disease evidenced by a rise in serum levels of PSA following local treatment with radical prostatectomy and/or radiation therapy [86]. 106 men participated in this multi-institutional study, with a median duration of 338 and 315 days of treatment for rosiglitazone and placebo, respectively. Based on serum PSA levels obtained every four weeks, rosiglitazone did not significantly increase the amount of time it took serum levels of PSA to double or increase the time before disease progression in men with prostate cancer. This trial does not support a role for rosiglitazone in recurrent prostate cancer, even if detected at the stage of biochemical, nonradiographic progression. Use of PPAR*γ* ligands in prostate cancer may require identification of susceptible tumor subtypes as well as consideration of intervention at earlier stages of disease.

### 2.5. Thyroid cancer

The thyroid gland is comprised of follicle cells, which produce thyroglobulin as well as synthesize and store thyroxine, triiodothyronine, and parafollicular C cells that produce calcitonin. Papillary and follicular thyroid cancers arise from follicle cells, comprising 80–90% of thyroid cancers. Differentiated thyroid cancers usually retain the ability to take up iodine, which serves as the basis for (1) radioactive iodine treatments to ablate residual or metastatic disease and (2) diagnostic screening via radioiodine scans [87]. These thyroid cancers also produce thyroglobulin, and elevated thyroglobulin levels following definitive therapy can signify recurrent disease.

Normal, benign, and malignant thyroid tissues express PPAR*γ*, with dysregulated expression in thyroid cancers [88, 89]. A PAX 8/PPAR*γ* rearrangement has been noted in half of all follicular thyroid cancers, in which the resulting protein has a loss of PPAR*γ* function [90]. Treatment of human thyroid cancer cells lines with thiazolidinediones inhibits cell proliferation and induces increased expression of markers of thyroid cell differentiation such as thyroglobulin, sodium-iodine symporter, thyroperoxidase, and TSH receptor [91–93].

These laboratory findings provided the basis for clinical investigation of the differentiating effects of PPAR*γ* ligands in thyroid cancer. Philips et al. conducted a pilot trial of rosiglitazone therapy in five patients with thyroglobulin-positive and radioiodine-negative thyroid cancer, a clinical scenario suggestive of dedifferentiation of the thyroid cancer [94]. After three months of rosiglitazone (4 mg/day for one month, then 8 mg/day for 2 months), thyroglobulin levels increased in 4 of 5 patients but only one subject had faint radioiodine uptake to delineate two lung metastases. Kebebew et al. also administered rosiglitazone (4 mg/day for 1 week, then 8 mg/day for an additional 7 weeks) to patients with recurrent or progressive papillary or follicular thyroid cancer who had elevated serum thyroglobulin levels and negative radioiodine scans [95]. In this open label phase II trial, 4 of 10 (40%) of the subjects had posttreatment radioactive iodine scans showing uptake in the neck (*n* = 3) and pelvis (*n* = 1). At the six month follow-up visit, serum thyroglobulin changes did not reveal a consistent pattern, with decreased (*n* = 2), increased (*n* = 5), and stable (*n* = 3) levels. There were no complete responses, defined as increased radioiodine uptake and decreased thyroglobulin levels. PPAR*γ* expression in pretreatment tumor samples by immunohistochemisty (formalin fixed, paraffin-embedded samples for eight patients) and real time quantitative RT-PCR (frozen samples for four subjects) did not correlate with the biological, biochemical responses. As noted in both reports, serum thyroglobulin level is somewhat problematic as a marker of differentiation in that increases could reflect re-differentiation/apoptosis as well as tumor progression. Future studies will also need to address heterogeneity of stage IV disease (e.g., some patients presented only with elevated thyroglobulin whereas others had diffuse metastases), dose level, duration of therapy, and intervention prior to development of metastases.

## 3. COMBINATION THERAPY

Despite the wealth of preclinical evidence for the anticancer effects of PPAR*γ* ligands in various types of cancer, thiazolidinediones appear largely ineffective as monotherapy agents for treating advanced, disseminated stages of cancer. However, cancer chemotherapy often involves the concomitant and/or sequential administration of multiple drugs in order to achieve maximal tumor cell kill and improved disease-free and overall survival. Combinatorial drug regimens may allow for additive if not synergistic effects which might also permit the use of lower dosages with decreased side effects. Preclinical studies show the potential benefits of combining PPAR*γ* ligands with other anticancer agents. For example, rosiglitazone treatment of A549 lung cancer cells increases the expression of PTEN (phosphatase and tensin homolog), which enhances the antiproliferative effects of the tyrosine kinase inhibitor gefitinib [96]. The combination of rosiglitazone and platinum-based cytotoxic drugs such as carboplatin and cisplatin synergistically inhibits the growth of A549 lung cancer cells relative to single-agent therapy [97]. Based on gene array analysis, rosiglitazone appears to mediate the downregulation of metallothioneins, heavy metal binding proteins involved in platinum drug resistance.

Lack of effect of PPAR*γ* ligand therapy may also relate to repression of ligand activation, such as by histone deacetylases (HDAC) which can form complexes with PPAR*γ* to repress gene transcription of specific PPAR*γ* target genes [98]. Removal of HDAC-mediated transcriptional silencing via HDAC inhibitors may allow activation of PPAR*γ*, as suggested by in vitro and in vivo studies of combined therapy of prostate cancer with HDAC inhibitors and PPAR*γ* agonists [85]. Inactivation of PPAR*γ* via phosphorylation could also occur in cancers with high levels of activity of mitogen-activated protein kinase [99], and coadministration of inhibitors of specific kinases might enhance the therapeutic potential of a PPAR*γ* agonist.

Novel treatment regimens combining pioglitazone with the COX-2 inhibitor rofecoxib and low-dose continuous chemotherapy drugs have been tested for efficacy in aggressive solid malignancies at advanced, progressive stages of disease. In a pilot study of 6 patients with advanced malignant vascular tumors that had progressed following surgery, radiotherapy, and/or chemotherapy, treatment with pioglitazone (45 mg/day), rofecoxib (25 mg/day), and metronomic trofosfamide (3 × 50 mg/day) resulted in 2 subjects with complete responses, 1 with a partial response, and 3 with stable disease [100]. The median progression-free survival was 7.7 months (range of 2 to 15 months), and the average treatment duration was 29.3, 21.7, and 23 weeks for pioglitazone, rofecoxib, and trofosfamide, respectively. Interestingly, one subject experienced regression of an extensive angiosarcoma of the facial skin with initiation of therapy with pioglitazone and rofecoxib alone. The Phase II trial of this treatment regimen in a cohort of patients with progression of previously treated metastatic melanoma (*n* = 19) or soft tissue sarcoma (*n* = 21) led to decreased tumor burden in 5 subjects and stabilization of disease in 6 subjects [101]. More recently, a multi-institutional randomized clinical trial of trofosfamide (50 mg TID) versus trofosfamide (50 mg TID) plus rofecoxib (25 mg/day) and pioglitazone (60 mg/day) of 76 patients with metastatic melanoma demonstrated superiority of metronomic chemotherapy combined with a COX-2 inhibitor and PPAR*γ* ligand, with 0% versus 9% progression-free survival [102]. Assessment of serum C reactive protein (CRP) levels in 48 subjects participating in this melanoma trial showed that those on the combined regimen had a greater than 30% decrease in this proinflammatory marker, suggesting that the anti-inflammatory effects of the drug combination may account for the improvement in progression-free survival. Interestingly, PPAR*γ* appears to suppress the proinflammatory potential of monocytes and macrophages [36, 103, 104], and thiazolidinediones have been shown to decrease CRP levels [105]. Another metronomic, low-dose chemotherapy regimen of either capecitabine (1250 mg/m^2^ twice daily for days 1–14 of 21 days) or temozolomide (50 mg/m^2^/d) combined with pioglitazone (60 mg/d) and rofecoxib (25 mg/d) was tested in patients who had developed recurrent high-grade gliomas (10 with gliobastoma, 4 with anaplastic astrocytoma) following chemotherapy, surgery, and/or radiotherapy, with 21.5% overall progression-free survival at six months (20% and 25% PFS for subjects with glioblastoma and anaplastic gliomas, resp.) [106]. By immunostaining the expression of COX-2, PPAR*γ*, and CD31 in these high-grade gliomas did not correlate with patient outcome; however, as recurrence was diagnosed by magnetic resonance imaging, tumor tissue obtained prior to disease progression might not accurately represent target gene expression in the recurrent tumors. The contribution of pioglitazone to the activity of these combination regimens is difficult to assess but may relate to anti-inflammatory effects as suggested by the CRP results in metastatic melanoma. PPAR*γ* ligands are also known to exert suppressive effects on angiogenesis in cancer [34] and on malignant vascular cells [107], which may also enhance the effectiveness of metronomic chemotherapy regimens for certain malignancies.

Combination with ligands to retinoid X receptor (RXR), the heterodimer partner of PPAR*γ*, may also augment the anticancer efficacy of PPAR ligand therapy. Treatment with RXR ligands alone appears efficacious in the treatment of certain malignancies, and bexarotene (Targretin) is a synthetic RXR-selective retinoid utilized in the treatment of refractory cutaneous T-cell lymphoma [108]. A Phase I study of bexarotene in patients with advanced cancer did not show objective tumor responses but indicated the potential for stable disease in 5 of 16 and 1 of 5 lung and head and neck cancer patients, respectively [109]. In a multi-institutional Phase II trial of bexarotene capsules (MINT or Targretin Monotherapy in non-small-cell lung cancer trial) in advanced non-small-cell lung cancer patients who had failed two or more prior chemotherapy regimens, bexarotene (400 mg/m^2^/day) did not meet the intended aim of a median survival of 6 months as third-line therapy; however, the subset of subjects with treatment-related hypertriglyceridemia (grade 1 to 4, 51%) and/or skin reactions such as rash, pruritis, and erythema (grade 1 to 4, 28%) had prolonged survival at one year of 30% and 34%, respectively, compared to 18% and 19%, respectively, of patients without these adverse events [110]. A role for bexarotene as monotherapy is also demonstrated in preclinical models of mammary carcinogenesis [111, 112]. A Phase II randomized trial of 200 or 500 mg/m^2^/day bexarotene in women with metastatic breast cancer refractory to endocrine therapy or chemotherapy showed clinical benefit in the form of complete/partial response and stable disease ≥6 months in 27 of 146 patients, with an objective response rate of 3 to 6% [113].

To evaluate RXR-PPAR*γ* ligand therapy for synergism in cutaneous T-cell lymphoma, a small uncontrolled study evaluated the addition of rosiglitazone to bexarotene monotherapy in four patients with stable or progressive cutaneous T-cell lymphoma, showing improvement in skin score (50%) and pruritus (75%) [114]. Preclinical studies of solid malignancies such as liposarcomas, breast, and colon cancers also provide support for the greater efficacy of combined regimens of PPAR*γ* and RXR ligands compared to monotherapy [26, 80, 115]. In addition to increased therapeutic benefits, combination therapy may also allow for lower doses and decreased toxicity.

Novel cancer agents that combine PPAR*γ* agonism with inhibition of other signaling pathways are also of interest. LY293111 is a diaryl ether carboxylic acid derivative that modulates multiple eicosanoid pathways, acting as a leukotriene B4 antagonist, 5-lipoxygenase inhibitor, and PPAR*γ* agonist [116]. Phase I trials have identified the maximally tolerated dose for continuous oral administration of LY293111, both as a single agent and in combination with irinotecan for solid malignancies [116]. Future clinical trials with correlative tissue analyses may provide insight into the specific contribution of PPAR*γ* agonism to the therapeutic potential of LY293111.

## 4. CANCER PREVENTION

PPAR*γ* may function as a tumor suppressor during early events of carcinogenesis. Thiazolidinediones or other PPAR*γ* ligands may have efficacy during early stages of cancer development, by targeting carcinogenesis prior to onset of initiating events that are no longer subject to PPAR*γ* repression. Indeed, mutant APC function and dysregulated *β*-catenin signaling in the APC^+/min^ mice model of colon carcinogenesis appear to abrogate responsiveness to PPAR*γ* activation [50]. Susceptibility to PPAR*γ* ligands is possibly limited to only certain premalignant conditions or individuals, and development of genomic strategies to properly apply this chemopreventive option in an otherwise heterogeneous high-risk population is needed.

In a phase II clinical trial of pioglitazone therapy for dysplastic leukoplakia, a precursor lesion to invasive oral cancer, pioglitazone administered for 3 months at 45 mg/day elicited a response in 70% of subjects (15/21) based on bidimensional measurements of the oral lesions [117]. Although the degree of dysplasia increased in three subjects, pioglitazone did not alter the degree of dysplasia in the majority of patients. These findings raise the possibility that PPAR*γ* ligands may prove efficacious for chemoprevention of oral cancer by decreasing the extent of leukoplakia. The lack of effect on dysplasia suggests that pioglitazone may not function as a differentiating agent for oral leukoplakia or at least within the time frame of a three month intervention.

## 5. SAFETY AND TOXICITY ISSUES

Even though the risk-benefit ratio of thiazolidinediones for cancer therapy seems favorable for advanced stages of disease, thiazolidinediones appear problematic as candidate agents for chemoprevention rather than for treatment of advanced, metastatic cancer. Use of thiazolidinediones in adjuvant therapy for cancer patients who are otherwise healthy but at increased risk for cancer recurrence would also raise concerns if administered on a long-term basis as for a chronic disease such as diabetes mellitus. Although generally well-tolerated, thiazolidinediones such as rosiglitazone and pioglitazone are associated with body weight gain as a result of increased adiposity and fluid retention, with the latter resulting in peripheral edema, anemia on the basis of hemodilution, and increased risk of developing congestive heart failure [118–120]. Controversy currently exists regarding the cardiovascular safety of rosiglitazone, with a recent meta-analysis of different treatment trials of rosiglitazone suggesting that rosiglitazone was associated with a significant increase in the risk of myocardial infarction and cardiovascular death [121]. However, other studies have not shown an increased risk of cardiovascular mortality, including the interim analysis of the Rosiglitazone Evaluated for Cardiac Outcomes and Regulation of Glycaemia in Diabetes (RECORD) trial, a prospective trial of rosiglitazone in type 2 diabetes mellitus [122, 123]. For postmenopausal women, rosiglitazone therapy has also been linked to osteoporosis and risk of fracture [124]. Such side effects are concerning for healthy individuals contemplating cancer risk reduction rather than treatment of disease. Defining the risks of thiazolidinediones will allow for avoidance of these drugs in subjects at higher risk for these associated complications.

The carcinogenic potential of thiazolidinediones and other PPAR*γ* agonists is another point of controversy [125, 126]. Upon review of the carcinogenicity of PPAR*γ* and dual PPAR*α*/*γ* agonists in development, the US Food and Drug Administration has recommended two-year carcinogenicity studies of new PPAR agonist drugs in rodents prior to initiation of clinical trials longer than six months [127, 128]. As discussed above, contradictory in vivo and in vitro findings may relate to specific animal models or molecular, genetic subtypes of certain cancers. Additionally, as diabetes and insulin resistance have been linked to higher risk for certain malignancies such as breast and colon cancer [72, 129, 130], thiazolidinediones might even exert preventive effects on carcinogenesis by ameliorating the chronic inflammation associated with dysregulated metabolism.

Postmarketing experience with pioglitazone and rosiglitazone has not shown significantly increased cancer risk with these drugs. The PROactive Study (PROspective pioglitazone clinical trial In macrovascular events), which randomized 5238 patients with type 2 diabetes mellitus and cardiovascular or peripheral vascular disease to pioglitazone or placebo for an average of 34.5 months, did not detect differences in the overall number of malignancies [131]. Interestingly, the pioglitazone treated group had more bladder tumors (14 versus 6) and fewer breast cancers (3 versus 11); however, the timing of the cancer diagnoses relative to pioglitazone use and the presence of confounding risk factors did not support a causal relationship to the study drug. Use of thiazolidinediones was associated with 33% decreased risk for lung cancer in a large retrospective database analysis of over 87,000 diabetic patients of 10 veteran affairs medical centers, without significant risk reduction for colorectal and prostate cancer [132]. Whether lung cancer is uniquely susceptible to thiazolidinediones or other PPAR*γ* agonists remains to be determined; preclinical studies suggest a potential role for PPAR*γ* ligand therapy in the management of this disease [133, 134]. In the preliminary report of a more recent analysis of this Veteran Affairs database, thiazolidinedione use also correlated with 41 to 55% reduced risk for head and neck squamous cell carcinoma [135]. Conversely, a smaller retrospective study of 1003 diabetic subjects in the Vermont diabetes information system database showed a possible association of cancer with use of thiazolidinediones and particularly rosiglitazone, based on self reported history of malignancy of any type [136]. Prospective studies with attention to confounding influences such as risk factors or timing and duration of drug use relative to cancer diagnosis are needed to investigate these possible associations and basis for the discrepant findings.

## 6. CONCLUSIONS

Based on the clinical experience to date, use of PPAR*γ* agonists in cancer therapy will likely require a targeted approach, with restriction to specific types of malignancies and/or stage(s) of disease susceptible to enhanced PPAR*γ* signaling. Studies of PPAR*γ* ligand therapy in cancer patients have focused largely on thiazolidinediones approved for treatment of type 2 diabetes mellitus, administered at the high end of doses tested for tolerability and insulin sensitizing effects. In phase II clinical trials, thiazolidinediones do not have significant activity in most cancers at advanced stages, which may relate to the extent and burden of disease and aggressive tumor phenotypes resistant to cytotoxic therapies. The lack of efficacy of thiazolidinediones in advanced, metastatic disease may relate to the use of noncytotoxic dose levels; alternatively, these drugs may not exert effects at late stages of cancer progression at any dose level. However, the demonstration of differentiating effects in some patients suggests that a subset of patients might benefit from PPAR*γ* ligand therapy. Thiazolidinediones and other PPAR*γ* agonists may ultimately prove effective in combination with standard cytotoxic agents or other molecularly targeted therapies. Interventions at earlier stages of disease, including premalignant or predisposing high-risk conditions, should also be considered. Therapy administered in the adjuvant setting, such as following surgical resection, or in situations of microscopic rather than measurable disease may also have greater efficacy. The use of PPAR*γ* ligands in earlier stages of cancer or cancer prevention will require additional research and/or drug development to address the questions regarding the carcinogenic potential and other adverse effects of these drugs. Genetic and molecular profiling of human cancers may in the future enable selection of a tumor subtype susceptible to PPAR*γ* agonists, as specific genotypes and patterns of nuclear receptor and cofactor expression might predict resistance or responsiveness to PPAR*γ* signal transduction.
